# Improving Behavioral-Based Safety Training in Using Verbal Commands Through a Theory-Driven and Feedback-Based Nonimmersive Virtual Reality Game: Development and Usability Study

**DOI:** 10.2196/48080

**Published:** 2024-03-12

**Authors:** Chukwudiebube Atagbuzia, Ean H Ng, Ganapathy Natarajan

**Affiliations:** 1 Oregon State University Corvallis, OR United States; 2 University of Wisconsin-Platteville Platteville, WI United States

**Keywords:** behavioral safety training, SERES framework, Reflection, Engagement, Choice, Information, Play, Exposition framework, gamification, gestalt laws of perception

## Abstract

**Background:**

The construction, chemical, aviation, medical, and health care industries have used serious games for safety training. To our knowledge, serious games have not been developed focusing on behavioral change to improve safety through the use of verbal commands and instilling players with heightened awareness of their spatial proximity to other people in their surroundings.

**Objective:**

We aimed to develop a theory-driven serious game for improving safety behavior using verbal commands and validate the implementation of the theoretical frameworks used for game development. The game developed, KitchenSpeak, was a first-person character (FPC) game where users respond to in-game prompts to use loud verbal commands when they are approaching another employee’s blind spot.

**Methods:**

In addition to using the SERES framework in guiding the general game design and development, and the Reflection, Engagement, Choice, Information, Play, Exposition (RECIPE) framework to inform the design of the game mechanics, we also applied gestalt laws of perception for graphic design to guide the design of the game’s user interface. We conducted 2 evaluative tests (alpha and beta) to collect end user and stakeholder feedback on the implementation of the theoretical frameworks, as well as to collect relevant information for full-scale implementation and a future validation study.

**Results:**

The alpha and beta tests had 8 and 40 participants, respectively. The alpha test results revealed that the theoretical frameworks were adequately applied; however, suggestions were also made to modify and improve the game. The beta test results suggested further improvements for the game design and found no differences in the perception of ease of play between participants with and without previous FPC gaming experience (*P*=.47; Kruskal-Wallis). Results suggested that the game met its design and theoretical requirements, and it would be easily playable by all players regardless of their previous experience in FPC games.

**Conclusions:**

A theory-driven and evidence-based FPC game titled KitchenSpeak was developed to teach the use of kitchen-speak terms in commercial kitchens. Evaluative tests were conducted to validate the implementation of the theoretical frameworks. Our main contributions are creating and validating game-based training to improve behavioral-based safety in the workplace and the incorporation of gestalt laws of perception for graphic design in the game’s user interface.

## Introduction

### Overview

In recent times, serious games have been used in industries and various occupations as training tools for employee safety. Serious games combine elements such as visual and auditory effects and the euphoria of completing challenges to facilitate user engagement to motivate the user to transfer gained knowledge to real-life experiences [1]. Serious games are effective tools to improve safety knowledge in the construction [2-8], chemical [9-11], medical and health care [12-15], and aviation [16-18] industries. However, to our knowledge, serious games have not been used in improving behavioral-based safety. In addition to acquiring safety knowledge, behavioral-based safety requires employees to repeat safe behavior over a period of time. Using verbal commands to alert fellow employees of their proximity is a safety practice that is important in high-traffic workplaces, where struck-by injuries can occur. Consistently using verbal commands is a behavior-based safety practice.

Verbal commands are used in various work settings, and this study used a commercial kitchen as a representative workplace to develop and validate our serious game. Verbal commands given in a commercial kitchen environment are generally called “kitchen-speak,” and they are widely used in commercial kitchens as a safety behavior aimed at reducing the risk of accidents due to collisions between employees. Existing methods to train employees to use verbal commands in the workplace using traditional training methods (lectures or videos with quizzes) have been ineffective. This study developed a theory-driven and feedback-based serious game for teaching and training employees to use verbal safety commands within a commercial kitchen.

### KitchenSpeak Game Overview

KitchenSpeak is a first-person character (FPC) game where users respond to in-game prompts to use loud verbal commands when they are approaching another employee’s blind spot. These verbal commands to alert other employees of their proximity are called kitchen-speak, the namesake of this game.

Commonly used verbal commands in a commercial kitchen include “corner,” “behind,” “hot,” “sharp,” and combinations of these commands, such as “hot corner” and “sharp behind.” In our game, the player will use the keyboard and mouse to navigate the game environment. Whenever a scenario to use a verbal command occurs, a pop-up text message will inform the player of the verbal command required to be yelled. Upon successful yelling of the command, the game will display another pop-up to inform the player. The player will not be able to proceed through the scenario until the proper command is verbally completed.

KitchenSpeak game consists of 4 stages, where players will progressively learn a new command in every stage while applying the commands learned in previous stages. The last stage is a free-roam stage, where players will have to use a combination of the commands they have learned without any pop-up window to indicate the place of use. The players should now be able to identify the safety situation and act without a prompt. In each stage, a minimum of 4 verbal commands are required for completion, and the players are allowed 1 manual override per stage to minimize frustration and game abandonment.

Prior to starting the game, the player will have to enable the microphone on their computer. The voice recognition module used in this game is Google’s speech-to-text application programming interface which is trained with Google’s speech recognition models. The players do not calibrate or train the speech recognition module.

### Significance of the Research

This study contributes to the body of knowledge on serious games by focusing on behavioral change to improve safety through the use of verbal commands and instilling the player with a heightened awareness of their spatial proximity to other people in their surroundings. Existing research in using serious games to improve safety has mainly focused on knowledge such as the proper use of tools [4,5] and proper procedures to perform high-risk tasks [19]. None of the existing serious games for safety focus on behavioral change and using verbal commands. The development and beta testing of the game from this study can be adapted to other training scenarios where behavioral change is needed and incorporate verbal commands as part of the gameplay.

## Methods

### Ethical Considerations

This study was reviewed by Oregon State University (OSU) institutional review board (IRB-2021-1174) and was determined to be exempt from review.

### Methods Overview

The SERES framework [20] for developing serious games was used to guide KitchenSpeak’s development. Nicholson’s [21] Reflection, Engagement, Choice, Information, Play, Exposition (RECIPE) framework was used to guide the design of the game mechanics within the design foundation stage of the SERES framework. KitchenSpeak’s user interface (UI) was designed according to the 6 gestalt laws of perception as proposed by Smith-Gratto and Fisher [22]. An effective UI is important for game development as it ensures that the game players engage with the game in the intended manner [23].

### SERES Framework

#### Overview

The five stages in the SERES framework are (1) scientific foundations, (2) design foundations, (3) development, (4) validation, and (5) implementation. This study applies the first 3 stages of the framework to KitchenSpeak game development. The last 2 stages of the framework involve evaluating the effectiveness of the training tool, which is planned for a follow-up study. The following subsections present a summary of how the first 3 stages of the SERES framework were applied, as well as the plan for implementing the last 2 stages.

#### Scientific Foundations

There were 4 aspects of this stage. First, the target audience was Marketplace West Dining Center (MWDC) student employees (84% of the MWDC employee population). Second, the outcome objectives were to increase the use and knowledge of kitchen-speak among student employees. Third, the theoretical basis was our hypothesis that kitchen-speak use and knowledge will increase among people who play the game. Fourth, the tool evaluation will be performed in future work prior to implementation.

#### Design Foundations

##### Game Mechanics

We designed the game mechanics according to the RECIPE framework for gamification. Please see the RECIPE Framework for Game Mechanics section for further details.

##### Design Requirements

We modeled the virtual environment after the real-world MWDC environment to motivate reflection and foster immersion. A plug-in was used to transcribe verbal inputs from the players. For easy access, KitchenSpeak was designed for a web browser platform. MWDC management advised that the game’s completion time should be between 5 and 10 minutes.

##### Game Authoring Tools

Based on the design requirements, the following software and services were used to develop the game: (1) Unity-3D game engine for WebGL, (2) Autodesk 3ds Max for object modeling, (3) Google’s speech-to-text plug-in for speech transcription, and (4) PHP database hosted on OSU servers for game data storage.

##### Trial Design

Two validation tests (alpha and beta tests) were used to validate the implementation of the theoretical frameworks.

#### Development

##### Genre

KitchenSpeak was modeled to be a FPC game. The FPC perspective camera model was used since it encourages user immersion more than games in the third-person camera perspective [24].

##### Content

**Figure 1:**
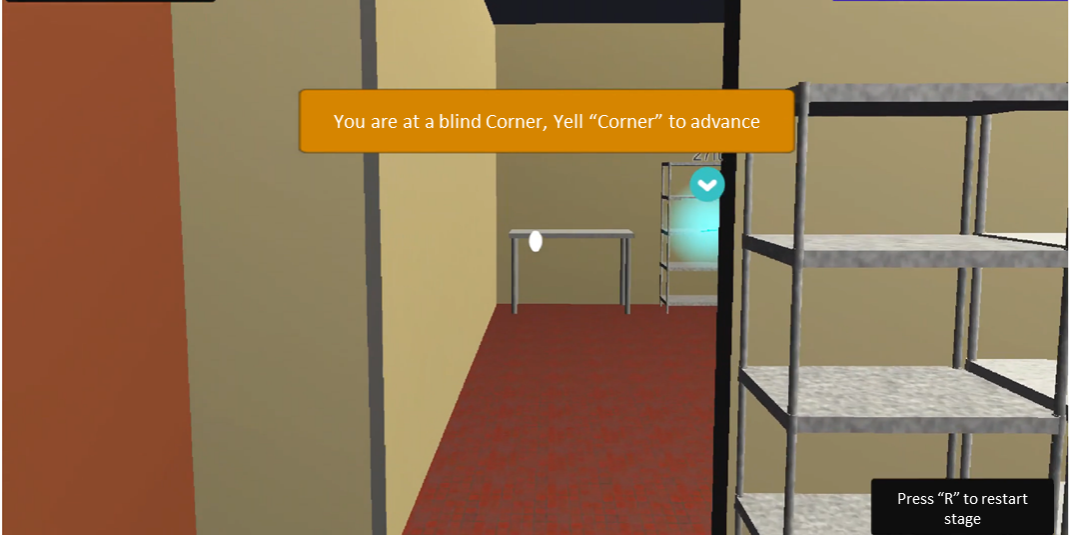
Prompt to use kitchen-speak in the Corner stage.

KitchenSpeak had 4 game stages: Corner, Behind, Hot Corner, and Free Roam stages. Each user must use kitchen-speak terms at least 3 times in the Corner and Behind stages, and at least 5 times in the Hot Corner and Free Roam stages. Each stage has a simple narrative where users are asked to complete tasks assigned by their supervisor. While navigating the virtual kitchen to complete tasks, the player is prompted to use kitchen-speak in blind areas. Figure 1 shows the “yell corner” prompt shown to a user as they approach a blind area. Learned kitchen-speak words cascade in each stage, that is, the Behind stage includes “corner” and “behind” kitchen-speak, the Hot Corner stage includes “corner,” “behind,” and “hot-corner,” and Free Roam includes all previously used kitchen-speak, with an additional “sharp-corner” kitchen-speak.

##### Rules

There are three general rules: (1) All game stages must be completed to complete the game. (2) Within the Corner, Behind, and Hot Corner stages, a player must use kitchen-speak terms when prompted; otherwise they do not advance through the blind area and cannot complete their assigned task (“enforced-use rule”; the Free Roam stage does not have this rule). (3) In the Corner, Behind, and Hot Corner stages, users are allowed to override the prompt to use kitchen-speak if they are stuck in the blind area for over 30 seconds; users are allowed a maximum of 3 overrides throughout the 3 game stages (override rule).

##### Visuals and User Experience

Gestalt laws of perception for graphic design were applied to guide the design of the UI elements. User experience was tested and modified through the alpha test.

#### Validation and Implementation

Validation and implementation, the last 2 elements of the SERES framework, will be performed in future work.

### RECIPE Framework for Game Mechanics

The six elements of the RECIPE framework were implemented in KitchenSpeak game mechanics as follows.

#### Reflection

**Figure 2:**
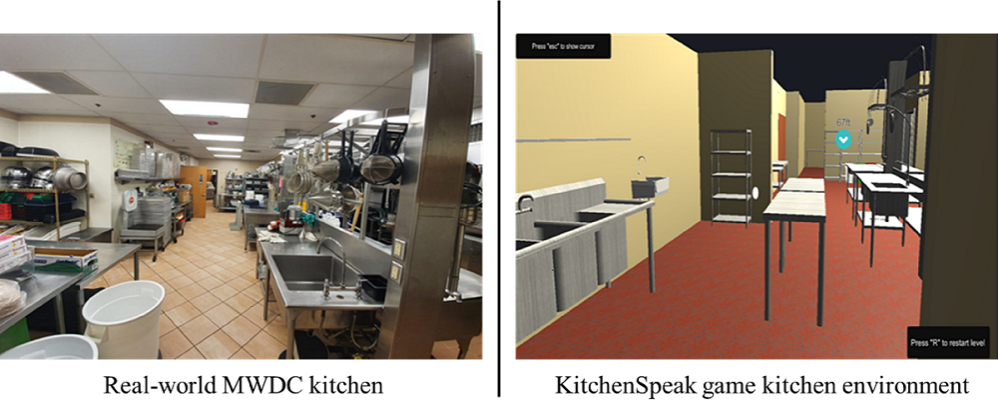
KitchenSpeak virtual environment and Marketplace West Dining Center (MWDC) kitchen.

This was implemented by modeling KitchenSpeak environment and activities after the MWDC kitchen environment, as well as the activities that occur within the MWDC kitchen. Figure 2 shows a scene of the virtual environment from KitchenSpeak compared to the MWDC environment.

#### Exposition

This was implemented by including simple narratives, such as “Your colleague John put some Teriyaki chicken into the oven, and it is now ready to be checked and served. Go and pick up the chicken from the oven.”

#### Choice

This was implemented by (1) allowing users to choose different navigating routes to complete an activity and (2) giving users the ability to choose when they want to complete a task. They may decide to free-roam within the game before completing the task.

#### Information

This was implemented by creating 3 tutorial stages that allow the user to practice the game controls before the main game stages. Informational texts highlighting the objectives of each stage are displayed at the start of each stage. A quick message is also shown to alert the player if they correctly use kitchen-speak, and if they have started, completed, or are in the process of completing a task.

#### Play

This was implemented by allowing players to restart each stage from within the game. If a user fails to complete certain tasks within a stage, the user is permitted to restart that stage an infinite number of times until they have completed the stage or feel satisfied with their progress. A player is always informed on their progress through the informational text during task progress and completion. Allowing the user to fail and restart within the learning environment creates an opportunity for the user to self-reflect and discuss their experiences with other players, thus improving long-term learning [25].

#### Engagement

This was implemented through interaction by including activities within the game that require the user’s response. The user is made to provide verbal input as a form of response to the kitchen-speak prompts and is asked to provide keyboard input while completing activities.

### Gestalt Laws of Perception for Graphics Design

**Figure 3:**
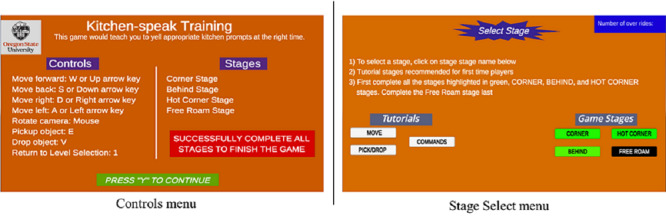
Controls and stage select menus.

Gestalt laws of perception for graphics design leverage the laws of perception to develop a comprehensible screen design that is aimed at aiding viewers to interpret and remember the presented materials [22,23]. The gestalt laws were applied to the UI elements within KitchenSpeak; however, the Controls menu and Stage Select menu in Figure 3 are used as examples to show how the laws were applied. The 6 gestalt laws of perception for graphics design applied in this game are as follows.

#### Figure-Ground Contrast

This law states that the figure and background must be distinct so that viewers can easily distinguish the figure from the background. In the Controls and Stage Select menus, the primary background color is dark orange (#FF8000) while the text color on the primary background is white (#FFFFFF). This color combination creates high contrast, which improves its legibility.

#### Simplicity

This law states that people simplify their perceptions based on previous experiences. The UI should be designed with simple and familiar designs so that viewers may easily understand it. In KitchenSpeak, unrelated information is shown in separate UI objects, while related information is shown in a single UI object.

#### Proximity

This law states that objects that are close together in space are generally perceived to be grouped together. Similar game UI objects were placed together to reduce the mental load that a player uses to identify game objects. In the Stage Select menu, the game stages and tutorial stages buttons are arranged in close proximity so that the player can easily identify that those buttons represent the game stages and training stages, respectively.

#### Similarity

This law states that objects that possess similar characteristics are generally perceived as a whole. KitchenSpeak game objects that were similar in function were designed to have similar appearances. In the Controls menu, the same background color was used for the subheadings Controls and Stages to signify that these 2 texts were subheadings and not parts of the instructions. In the Stage Select menu, the game stage buttons are made the same color apart from the Free Roam stage button. The Free Roam stage button turns green after the player successfully completes the 3 other stages.

#### Symmetry

This law states that objects with an unbalanced symmetry are perceived to be incomplete and could be distracting to viewers. In the Controls menu, the white vertical line between the Controls and Stage subheadings was designed to create a symmetrical design in the UI. Symmetry was also applied in the Stage Select menu to guide the arrangement of the game stage buttons.

#### Closure

This law states that open shapes are perceived as incomplete. Important figures should have closed shapes so that viewers do not have to be distracted from trying to close the shape. In the Controls menu, the message “Successfully complete all stages to finish the game” conveys information that is different from the information that the other UI objects convey. As a result, its UI object was designed to have a closed red rectangular figure that distinguished it from the other UI objects, thus reducing ambiguity and making the information easier to read.

### Trial Design

Two evaluation tests (alpha and beta tests) were conducted. The alpha test was conducted as a qualitative test in the form of an open-ended discussion where participants played KitchenSpeak version 1.0 and were asked to provide their verbal feedback on their gameplay experiences. Our focus for the alpha test was the game content, navigation, UI, speech recognition, realism of environment, narrative, choice, information, and the restart and override rules.

The beta test was conducted by releasing KitchenSpeak version 2.0 to MWDC staff and collecting their (1) game completion times, (2) perception of ease of play, (3) gameplay engagement, and (4) experience with bugs and glitches during gameplay. A 5-point Likert scale survey was used to collect user perception of ease of play, while a survey with predetermined questions was used to collect users’ experience with bugs and glitches.

## Results

### Alpha Test Results

We recruited 8 participants, including 5 MWDC management staff and 3 nonmanagement staff with varying gaming experience, who were recruited by MWDC management. Participants played the game one after another with the debugging window opened to observe how the speech recognition model transcribed the participant’s speech input. The results from this qualitative test are provided in Table 1.

**Table 1:** Results from the alpha test.

Aspects of the game	Results	Suggestions
Realism of game environment	Participants deemed the game environment design was similar in appearance to the MWDC^a^ kitchen.	Include more details to reflect real-world execution of activities, for example, while a player is picking up a hot pot from the gas oven top, they should turn off the gas and use heat-resistant gloves.
Content and narrative	Game narratives were perceived as short, but it was explained that they were intentionally kept short to avoid confounding the narrative with the game’s purpose. All participants agreed that the content and narrative were sufficient.	No suggestions for improvement.
Navigation	Difficulty in navigating for participants with no previous FPC^b^ gaming experience.	Limit the vertical camera viewing angle such that it cannot go above the game character’s horizontal line of sight. That is, FPC cannot look up, but only downwards.
Information	Instructions in the Stage Select menu were not easy to understand, and participants did not know how to start playing a stage.	Include descriptive instructions on how participants could access game stages.
UI^c^	UI elements were without clutter, simple, and easy to understand.	No suggestions.
Speech recognition usability	Single-word kitchen-speak (ie, “corner” and “behind”) was transcribed correctly, but the “hot corner” phrase was often transcribed as “hot burner “and “hot porter.”	Include “hot burner “and “hot porter” phrases into a similar keyword dictionary such that they will be accepted as correct alternatives to “hot corner” when transcribed.
Choice	Participants enjoyed the liberty to explore the game environment while completing tasks. One participant mentioned that exploring the kitchen environment reminded them of the MWDC kitchen.	No suggestions.
Game rules	Two overrides per game session were too little given the accuracy of the speech recognition module. Participants agreed that the restart rule was good as it would allow users to practice within each stage.	Increase the allowable number of overrides per session to 3.

^a^MWDC: Marketplace West Dining Center.

^b^FPC: first-person character.

^c^UI: user interface.

### Beta Test Results

#### Overview

Feedback from the alpha test was used to release version 2.0, which was used for the beta test. The beta test had 40 participants; 34 of them played and responded to the FPC experience and the ease of play surveys, and 32 responses were valid. A total of 21 participants indicated they were in the 15-to-24-year age group, while 11 participants did not respond to the age question; 7 participants were male, 11 were female, 1 was nonbinary, and 13 did not indicate their gender. The age group and gender proportions were representative of the student population in MWDC. A total of 13 participants responded to the Bugs and Glitches questionnaire.

#### Ease of Play

We looked at the difference in perception of ease of play between participants with previous FPC experience and those without previous FPC experience. Table 2 shows the summary of the ease of play results. The average ease of play of all 32 participants was 3.91 (SD 1.12). There was no significant difference in the user’s perception of ease of play between players who had previous FPC experience and players who had no previous FPC experience at a .05 significance level.

**Table 2:** Examining perception of ease of play.

Group	Ease of play score^a^
All participants, mean (SD)	3.91 (1.12)
Had previous FPC experience, mean (SD)	4 (1.06)
No previous FPC experience, mean (SD)	3.63 (1.30)
Kruskal-Wallis *P* value	.47

^a^Measured on a 5-point Likert scale.

#### Game Completion Time

We evaluated the average time it took a player to complete game stages and the difference in completion time between participants with FPC gaming experience and no FPC gaming experience. Table 3 shows a summary of the game completion time test. The Kruskal-Wallis test for game completion times indicated that there was a significant difference in the median time it took to complete the game between users with FPC experience and users without FPC experience.

**Table 3:** Statistical test results for completion times.

Group	Game completion time (seconds), mean (SD)
All participants (n=32)	369.94 (164.33)
Had previous FPC experience (n=24)	325.95 (129.41)
No previous FPC experience (n=8)	501.93 (195)
Kruskal-Wallis *P* value	.005^a^

^a^Significant at 5% significance level.

#### Gameplay Engagement

At least 160 prompts were shown to all 32 players (minimum of 5 kitchen-speak prompts per player). We measured disengagement by the ratio of the number of times a user did not use kitchen-speak when prompted to the minimum number of times a prompt to use kitchen-speak was shown in the Free Roam stage (5 prompts). The results revealed that out of more than 160 prompts that were shown to all 32 players, 11 (6.9%) prompts did not receive valid user responses, while at least 149 (93.1%) prompts received valid user responses.

#### Bugs and Glitches

A total of 13 participants completed the Bugs and Glitches questionnaire. A total of 8 reported no issues, 5 had bugs in the Free Roam stage, and 2 had speech recognition issues. One participant found the game realistic. While the speech recognition module was adjusted to reduce recognition errors after the alpha test, issues with speech recognition were still expected.

## Discussion

### Overview

As part of this discussion, we will examine implications of this study and implications for future game developers based on our results. The major topics of discussion will be the frameworks used for game development, the results of the alpha and beta tests, and the development of serious games for behavioral safety.

### Frameworks for Game Development

The frameworks used for our game design were SERES and RECIPE frameworks. In addition, we used gestalt laws of perception to design an effective UI for a serious game. In evaluating our implementation of the RECIPE framework, we found that evaluating player engagement was challenging. We measured engagement through the lack of disengagement, and we found our approach to be lacking. Engagement is a multiconstruct measurement [26] and as such must use a triangulation method using more than 1 input. For future game development, developers should consider including multiple measurements for engagement during the design phase.

Effective UI design can improve the gaming experience for inexperienced players, and the gestalt laws of perception can bridge the gap for game developers in designing an effective UI. Previous gaming experience affects user experience as inexperienced players report less positive experiences than experienced players [27]; however, effective UI design can be used to improve user experience and aid effective learning in electronic learning platforms [23]. Gestalt laws of perception for UI design are effective because they provide a holistic perspective that considers humans’ perception and interpretation of information [23]. We applied gestalt laws of perception to design the UI and evaluated the UI’s usability. The alpha test results showed that the UI design was without clutter, simple, and the information was easy to understand. We encourage serious game developers to incorporate the laws of perception to improve their game design by reducing clutter and improving information retention with players.

### Alpha and Beta Testing

The alpha tests showed us the importance of receiving feedback to improve the game. Including the players, the most important stakeholders, is key to successful game deployment. In our case, we used a qualitative method that provided rich feedback for version 2. Our methods here show that a full testing regime that includes quantitative methods such as A/B testing is not always required. Receiving qualitative feedback to make quick improvements is an effective and agile methodology we suggest for serious game developers.

Our analysis of game completion time showed players without FPC gaming experience completed the game significantly more slowly than those with prior experience. Even though we had a tutorial for inexperienced players, the difference in completion time results was significant. This shows that additional training is required. Players with no prior FPC experience must be eased into gameplay by observing player behavior and receiving real-time feedback. Failure to ease players into the game may cause frustration and game abandonment.

In terms of bugs and glitches, voice recognition was an area of interest. There was variability due to factors such as background noise levels and user-specific characteristics. User accent and voice pitch are factors that will inherently affect the accuracy of a speech recognition module. Accent recognition is a problem of particular interest that requires further exploration.

KitchenSpeak game used the Unity3D WebGL. We used Unity3D’s profiling tool to review game assets and memory usage. The results showed that CPU memory was mostly used by rendering game object textures and audio data. To improve overall game performance, similar textures were applied to different game objects to reduce the number of times a type of texture was rendered. We encourage game developers to consider this aspect while developing a game and to use this technique to promote game performance.

### Serious Game for Safety Behavior

Prior work in serious games for safety mainly focused on safety knowledge training. There are a few challenges in developing a serious game for behavior change from the safety behavior theoretical perspective. Safety behaviors are defined as acts that can be observed by other employees. Existing theories on changing safety behaviors are rooted in changing values, attitudes, and motivations, and emphasize providing consequences for not practicing the behavior [28].

The first challenge we encountered was finding the balance between providing sufficient exposition in the game to affect the value, attitude, and motivations of the players and managing the length of the gameplay. Our current approach to exposition is a pop-up text box to provide context. If the pop-up window is text-heavy or if we use too many consecutive pop-up windows, based on our own gameplay experience, it can lead to players skipping the pop-up windows or abandoning the game. We have opted to minimize the text in exposition, but have the player repeat the same behavior (verbal commands) multiple times for each play, each with a different exposition.

Second, the literature on safety behavior states that behavior must be observable by other employees; we included nonplayer characters (NPCs) into the scenes within our game. For future work, we plan to collect the gameplay information of actual players and create NPC movements based on actual players’ gameplay data to increase the realism of the NPCs. In either case, we are still looking into methods to assess whether the NPCs create the effect of observers.

Third, in terms of consequences to players when they did not perform the safety behavior, we considered adding consequences, and we evaluated two potential approaches. One is to create and display virtual scenes of accidents such as the spilling of hot food content after the collision and another is to use a pop-up window to inform the player that an accident had happened due to them not providing verbal commands. We decided against the pop-up window as we used it for exposition and did not want to overload the players and have them ignore it. Due to time constraints, we were not able to implement the actual scene of the accident for this study.

For safety behavior, using verbal commands consistently throughout an entire work shift is a relatively new concept to many employees who have never worked in a commercial kitchen. The behavior change we initiated through this study can serve as the baseline for future work to assess whether a change in value or attitude or motivations, a need for observers, and a need for consequences are necessary in a serious game to initiate behavior change.

### Conclusion and Future Work

Serious games and other e-learning modules could be used as a cost-effective method for training as employers do not have to deal with the costs of face-to-face in-person training [29]. A serious game is a game that achieves an additional goal that is not for entertainment purposes [30]. By this definition, KitchenSpeak is a serious game. This study aimed to advance the serious games research field by developing a game aimed at changing behavior, specifically, instilling the behavior of consistently using verbal commands in blind areas within high-traffic workplaces.

KitchenSpeak development was successful; however, a new study should evaluate its effectiveness in improving the use of verbal commands and its effect on accidents that lead to struck-by injuries. In order to assess how effective KitchenSpeak is in enhancing the use of verbal commands, we need to compare the frequency of verbal command terms used in a commercial kitchen where the game is deployed to the frequency of kitchen-specific terms used in a commercial kitchen where the game is not deployed. To evaluate KitchenSpeak’s effect on accidents that lead to struck-by injuries, we will compare safety records on struck-by injury frequency before and after the game’s deployment in a commercial kitchen.

In this study, we discussed and validated the implementation of 3 theoretical frameworks that were used to develop the KitchenSpeak serious game for commercial kitchen safety. A serious game’s effectiveness is improved by incorporating theoretical concepts and principles that intend to achieve the desired results [30]. While theoretical concepts and principles serve as relevant tools for guiding serious game development, their effectiveness is linked to their success in application and implementation. In addition to testing the effectiveness of a serious game in achieving its desired outcomes, developers may also evaluate the implementation of their foundational theoretical principles, as they may provide relevant information on the cause of their game’s effectiveness or ineffectiveness.

## Abbreviations

FPC: first-person character
MWDC: Marketplace West Dining Center
NPC: nonplayer character
OSU: Oregon State University
RECIPE: Reflection, Engagement, Choice, Information, Play, Exposition
UI: user interface

## Data Availability

The data sets generated during and analyzed during this study are available from the corresponding author on reasonable request.
